# PCAT6 may be a new prognostic biomarker in various cancers: a meta-analysis and bioinformatics analysis

**DOI:** 10.1186/s12935-021-02079-4

**Published:** 2021-07-12

**Authors:** Song-bo Shi, Qing-hao Cheng, Shi-yi Gong, Ting-ting Lu, Shi-fang Guo, Shao-ming Song, Yu-ping Yang, Qi Cui, Ke-hu Yang, Yao-wen Qian

**Affiliations:** 1 Gansu Provincial Hospital, 204 Dong gang West Road, Cheng guan District, Lanzhou, China; 2 Institution of Clinical Research and Evidence-Based Medicine, The Gansu Provincial Hospital, Lanzhou, China; 3 Evidence-Based Medicine Center, School of Basic Medical Sciences, Lanzhou University, No. 222, Tian shui South Road, Cheng guan District, Lanzhou, 730000 China; 4 Key Laboratory of Evidence-Based Medicine and Knowledge Translation of Gansu Province, Lanzhou, China; 5 School of Clinical Medicine, Ningxia Medical University, Yinchuan, China; 6 The First Clinical Medical College of Lanzhou University, Lanzhou, China

**Keywords:** Cancers, lncRNA PCAT6, Meta-analysis, Prognosis

## Abstract

**Background:**

LncRNA prostate cancer-associated transcript 6 (PCAT6) has been reported to be dysregulated in several cancers and is associated with tumor progression. Here, we have performed a meta-analysis to assess the general prognostic role of PCAT6 in malignancies.

**Methods:**

Four public databases (Embase, Pubmed, Web of Science, Cochrane Library) were used to identify eligible studies, then data was extracted and associations between prognostic indicators and clinical characteristics were combined to estimate hazard ratio (HR) or odds ratio (OR) with a 95% confidence interval (CI). Publication bias was measured using the Begg's test, and the stability of the combined results was measured using sensitivity analysis. Subsequently, results were validated using Gene Expression Profiling Interactive Analysis (GEPIA) and the National Genomics Data Center (NGDC).

**Results:**

Ten studies were considered eligible for inclusion. In total, 937 patients and eight types of cancer were included. Our results revealed that overexpression of PCAT6 was significantly associated with a shorter OS (HR = 1.82; 95% CI, [1.40, 2.38]; *P* < 0.0001) and progression-free survival (PFS) (HR = 1.66; 95% CI, [1.22, 2.25]; *P* < 0.0001) in cancer patients, and that PCAT6 overexpression was significantly associated with individual tumor clinicopathological parameters, including TNM stage (OR = 0.29; 95% CI, [0.09, 0.94]; *P* = 0.04), gender (OR = 1.84; 95% CI, [1.31, 2.59]; *P* = 0.0005), and whether the tumor was metastatic (OR = 5.02; 95% CI, [1.36, 18.57]; *P* = 0.02). However, PCAT6 overexpression was not correlated with patient age and tumor differentiation. PCAT6 expression was significantly up-regulated in four types of cancer, which was validated using the GEPIA cohort. Combining OS and disease-free survival (DFS) of these four types of cancer revealed a shorter OS and DFS in patients with PCAT6 overexpression. PCAT6 expression in various types of cancer was also validated in NGDC. A total of eight cancers were analyzed and PCAT6 was highly expressed in all eight cancers. Further functional predictions suggest that PCAT6 is correlated with tumor prognosis, and that PCAT6 may be useful as a new tumor-specific marker.

**Conclusions:**

LncRNA PCAT6 is highly expressed in multiple cancer types and its upregulation was significantly associated with patient prognosis and poorer clinical features, thereby suggesting that PCAT6 may be a novel prognostic factor in multiple cancer types.

## Introduction

Cancer is ranked globally as a leading cause of death and a significant barrier to increasing life expectancy. In 2020, there were an estimated 19.3 million new cancer cases and 10 million cancer deaths worldwide, and the incidence is expected to increase to a projected 28.4 million new cases by 2040 [1]. The American Cancer Society estimated that there will 1,806,590 new cancer cases in the United States in 2020, resulting in an estimated 606,520 cancer-related deaths [2]. In China, an estimated 4.51 million cancer cases and 3.04 million cancer-related deaths were reported in 2020 [3]. Despite rapid developments in medical technology and the increasing number and sophistication of treatment methods for tumors, the overall prognosis of cancer remains poor. One of the main reasons for this poor overall prognosis is the lack of specific markers for tumor diagnosis; thus, when a patient is diagnosed with cancer, it has usually progressed to the middle and late stages [4]. As a consequence of this delay in diagnosis, the optimal opportunity to treat cancer is usually lost. Hence, the search for specific tumor markers is of great importance in cancer diagnosis. Biological markers (‘Biomarkers’) play an increasingly important role in determining risk and detecting existing early-stage cancers or precancerous lesions [5]. These biomarkers can be biological indicators of normal biological processes, pathogenic processes, or pharmacological responses to therapeutic interventions.

Long non-coding RNAs (lncRNAs) are mRNA-like transcripts ranging in length from 200 nucleotides (nt) to 100 kilobases (kb) with no apparent open reading frame (ORF) [6, 7]. Although initially considered as transcriptional noise, studies have revealed that lncRNAs are involved in a variety of pathophysiological processes, especially cancer, and an increasing number of lncRNAs are often aberrantly expressed in cancer [8–10]. Therefore, lncRNAs may be useful as biomarkers for early tumor detection and prognostic monitoring. In recent decades, research on lncRNAs has focused on the dysregulation of lncRNAs involved in the pathophysiological processes of human diseases, including cancer [11]. It is now known that lncRNAs play a role in a variety of biological processes, including autophagy, myocardial infarction, cellular senescence, apoptosis, cancer cell metastasis, and resistance to chemotherapeutic agents. In addition, they can modulate the regulation of gene expression through a variety of mechanisms, including epigenetic modification, selective splicing, nuclear import, precursors to small RNAs, and even as regulators of mRNA modifiers or decoy elements [12–16].

The discovery of lncRNAs has enriched our understanding of the diversity and complexity of genetic information. From a mechanistic perspective, studying lncRNAs is crucial for the development of new biomarkers and effective therapeutic targets for cancer patients. Our understanding of the role of lncRNAs in the progression and suppression of multiple tumors has resulted in novel opportunities to improve the diagnosis and treatment of malignancies [17, 18]. Meta-analyses revealed that HOXD-AS1[4], PODXL [19], and TP73-AS1 [20] were associated with the clinicopathological characteristics and prognosis of tumors. LncRNA prostate cancer-associated transcript 6 (PCAT6), which was first identified in keratinocytes, indirectly activates the Wnt/β-catenin pathway by interacting with KLHL12 in cervical cancer cells [21]. Using LncRNA microarrays, PCAT6 was further confirmed to be the most up-regulated lncRNA expressed in cancer tissues, and it was revealed to be significantly associated with prostate cancer metastasis, induced-colony formation, and the proliferation of keratin-forming cells in prostate cells [22]. In addition, PCAT6 has been shown to be aberrantly expressed in osteosarcoma [23], ovarian [24], lung [25], colorectal [21], cervical [26], and pancreatic ductal cancers [27], thereby suggesting that PCAT6 is associated with the clinicopathological characteristics and prognosis of tumors.

Numerous studies have reported that aberrant expression of PCAT6 in various types of cancer correlates with pathological features, such as tumor size, metastasis, degree of differentiation, TNM stage, and prognosis [24–26]. To provide better clinical guidance to clinicians, in the present study we have performed a meta-analysis of the existing literature to investigate the relationship between PCAT6 and clinicopathological features and patient prognosis [28].

## Materials and methods

### Search strategy

This meta-analysis was conducted and reported according to the Preferred Reporting Items for Systematic Reviews and Meta-Analysis (PRISMA) guidance. Four databases, Embase, Pubmed, Web of Science, and Cochrane Library, were searched from database creation to 1st March 2021 with no language restrictions. For this search, Medical Subject Headings (MESH) terms and free terms were combined. Our search terms were: ("Neoplasms" OR "Carcinoma" OR "Prognosis" OR "Diagnosis" OR "Survival") AND ("Prostate cancer-associated ncRNA transcript 6" OR "PCAT6"). Two reviewers independently reviewed the database search strategies and discussed the results to ensure accuracy and consistency [29].

### Inclusion and exclusion criteria

The inclusion criteria were as follows: (1) PCAT6 expression in cancer patients was examined in tumor tissues by qRT-PCR or RNA-seq; (2) patients had a clear diagnosis of cancer and the relationship between PCAT6 and survival information or clinicopathology was described; and (3) hazard ratio (HR) for overall survival (OS) and progression-free survival (PFS) were provided or could be calculated from survival curves.

The exclusion criteria were as follows: (1) reviews, case reports, letters, editorials, conference reports, and in vitro or in vivo experiments; and (2) it was not possible to extract the data or to perform valid operations on the extracted data.

### Literature selection and data extraction

Two researchers (Cheng and Shi) independently searched and selected the literature based on the above-mentined criteria and extracted the following information: (1) name of first author; (2) publication year; (3) country where the study was performed; (4) types of cancer; (5) number of patients; (6) detection methods; (7) cut-off criteria; (8) clinical parameters; (9) OS data; and (10) PFS data.

### Data extraction and quality assessment

Two reviewers (Cheng and Shi) independently extracted the data and assessed the quality of included studies. Disagreements were resolved by consultations with the third reviewer [30]. The quality of the studies was assessed using the Newcastle–Ottawa scale (NOS). The assessment scale consisted of three domains: selection of study groups; comparability; exposure (case–control study); or outcome (cohort study). The maximum total score was nine points; one star represented one point. The scores were divided into low quality (0–3), medium quality (4–6), and high quality (7–9), which was based on the total score.

### Public data and tools

Gene Expression Profiling Interactive Analysis (GEPIA; http://gepia.cancer-pku.cn), which is based on The Cancer Genome Atlas (TCGA; http://cancergenome.nih.gov/) [31], was performed to verify the abnormal expression of PCAT6 among cancer tissues and to match TCGA normal and GTEx data among various neoplasms (cut-off, *P* < 0.01). Survival plots of the correlation between PCAT6 expression and OS and disease-free survival (DFS) were retrieved as Kaplan–Meier curves based on different cancer datasets. To further validate our results, PCAT6 expression in various cancer types was retrieved from the Database Resources of the National Genomics Data Center (NGDC; https://bigd.big.ac.cn) [32].

### Statistical analysis

RevMan (Version 5.4) software was used to calculate the pooled ORs and HR with 95% CIs. The *I*^*2*^ value and the χ2 -test were used to assess heterogeneity. A fixed-effects model was used if the heterogeneity was low (*P * >  0.1, *I*^*2*^  ≤  50%), and a random effect model was used if the heterogeneity was high (*P*  <  0.1, *I*^*2*^  >  50%.). A *P*  <  0.05 was considered statistically significant. Engauge Digitizer (Version 10.0) software was used to extract survival data from the Kaplan–Meier curves. Begg’s tests and sensitivity analyses were performed using Stata SE12.0 software. Begg’s tests were used to assess potential publication bias, while sensitivity analyses were used to examine sources of heterogeneity and the stability of the results.

## Results

### Literature screening

**Fig. 1:**
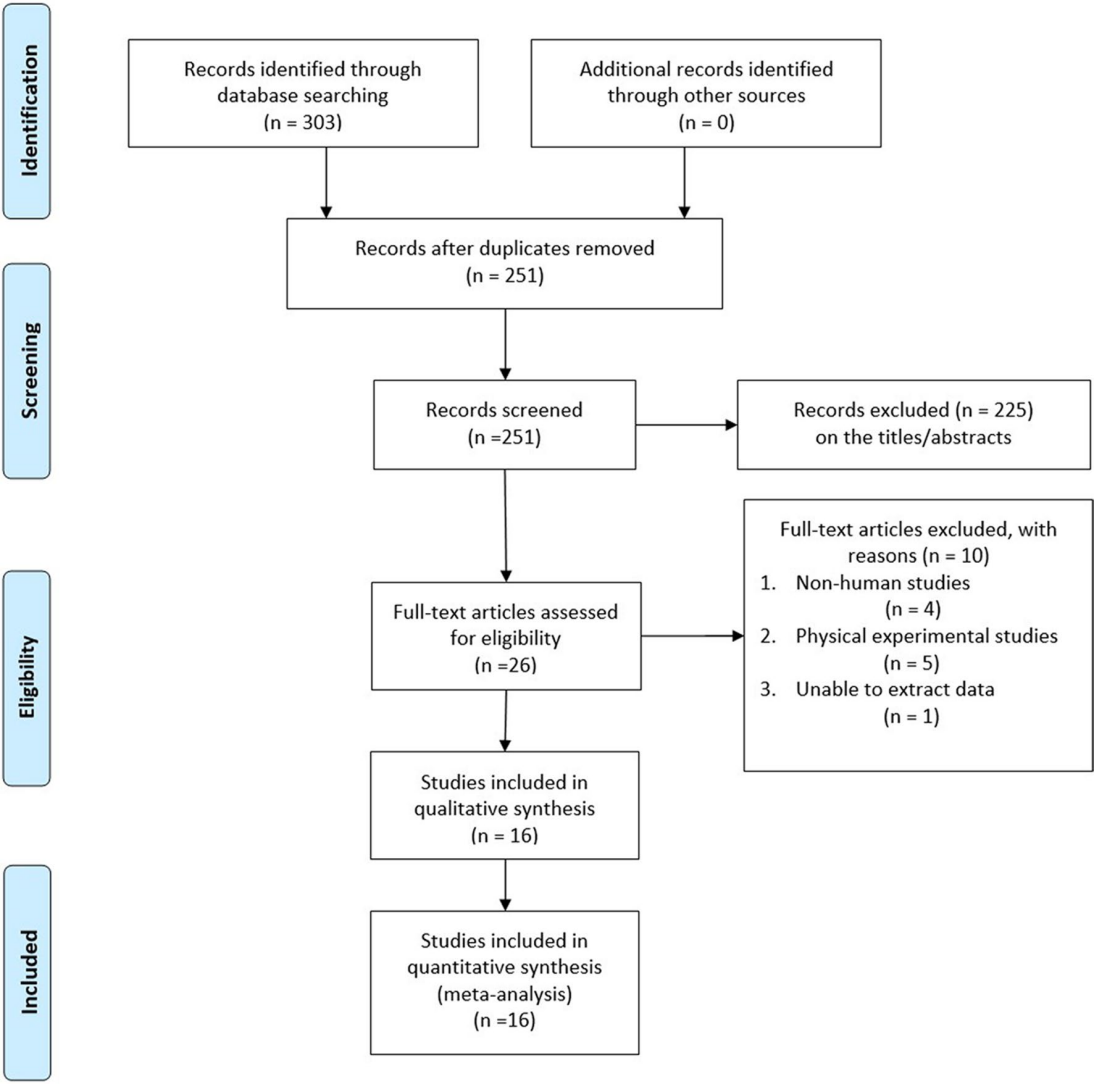
Flow chart of the literature search and selection process

We searched four databases to retrieve a total of 92 studies (Pubmed = 30, Cochrane Library = 0, Web of Science = 30, Embase = 32). A total of 58 duplicate articles were removed using Endnote X9. After further examination of the titles and abstracts, 10 irrelevant studies were excluded. Finally, the full text of 24 articles was carefully examined. One article was excluded because data could not be extracted, seven articles were excluded because patient information was missing, and five articles were excluded because the experiments were performed on cells only. Finally, a total of 10 articles [17, 21, 23–27, 33–35] were included in this study. Information concerning the selection process is provided in Fig. 1.

### Study characteristics and quality assessment

The characteristics of the 10 included studies are listed in Table 1. One study [34] had a score of seven and was considered of high quality, eight studies [17, 21, 23–27, 33] had a score of six, and one study [35] had a score of five, and were considered of moderate quality. These studies were published in 2017–2020 with sample sizes ranging from 43 to 114. In all ten studies, 937 patients were divided into PCAT6 high and low expression groups based on the results obtained by RT-qPCR. The types of cancer included osteosarcoma, cervical cancer, lung cancer, pancreatic ductal adenocarcinoma, colorectal cancer, bladder cancer, gastric cancer, and ovarian cancer. In total, eight studies reported OS results, four studies reported PFS results, and all 10 included studies reported results for clinicopathological parameters.

**Table 1:** Characteristics of eligible studies in this meta-analysis

ID	Year	Country	Sample size (high/low)	Cancer type	Method	Outcomes	HR availability	Follow-up months (mouth)	NOS Score
Zhu 2020 [35]	2020	China	60 (30/30)	Osteosarcoma	qRT-PCR	OS, PFS, CP	K-M curve	60	5
Lv 2019 [27]	2019	China	114 (58/56)	Cervical cancer	qRT-PCR	OS, PFS, CP	K-M curve	60	6
Wan 2016 [18]	2016	China	58 (27/31)	Lung cancer	qRT-PCR	OS, CP	K-M curve	60	6
Wang 2020 [28]	2020	China	67 (37/30)	Pancreatic ductal adenocarcinoma	qRT-PCR	OS, CP	K-M curve	60	6
Wu 2019 [26]	2019	China	73 (37/36)	Colorectal cancer	qRT-PCR	OS, CP	K-M curve	60	6
Wu 2020 [23]	2020	China	106 (70/36)	Osteosarcoma	qRT-PCR	OS, PFS, CP	K-M curve	60	6
Zhang 2020 [34]	2020	China	106 (53/53)	Bladder cancer	qRT-PCR	OS, PFS, CP	K-M curve	150	7
Xu 2018 [33]	2018	China	72 (36/36)	Gastric cancer	qRT-PCR	OS, CP	K-M curve	100	6
Shi 2018 [25]	2018	China	60 (31/29)	Lung cancer	qRT-PCR	CP	K-M curve	–	6
Kong 2019 [24]	2019	China	42 (18/24)	Ovarian cancer	qRT-PCR	CP	K-M curve	–	6

*OS* overall survival, *PFS* progression-free survival, *CP* clinicopathological parameters, *NOS* Newcastle–Ottawa Scale, *K-M curve* Kaplan–Meier curve, *qRT-PCR* quantitative real time polymerase chain reaction, -, not available or invalid

### Meta-analysis

#### Association between PCAT6 expression and prognosis

**Fig. 2:**
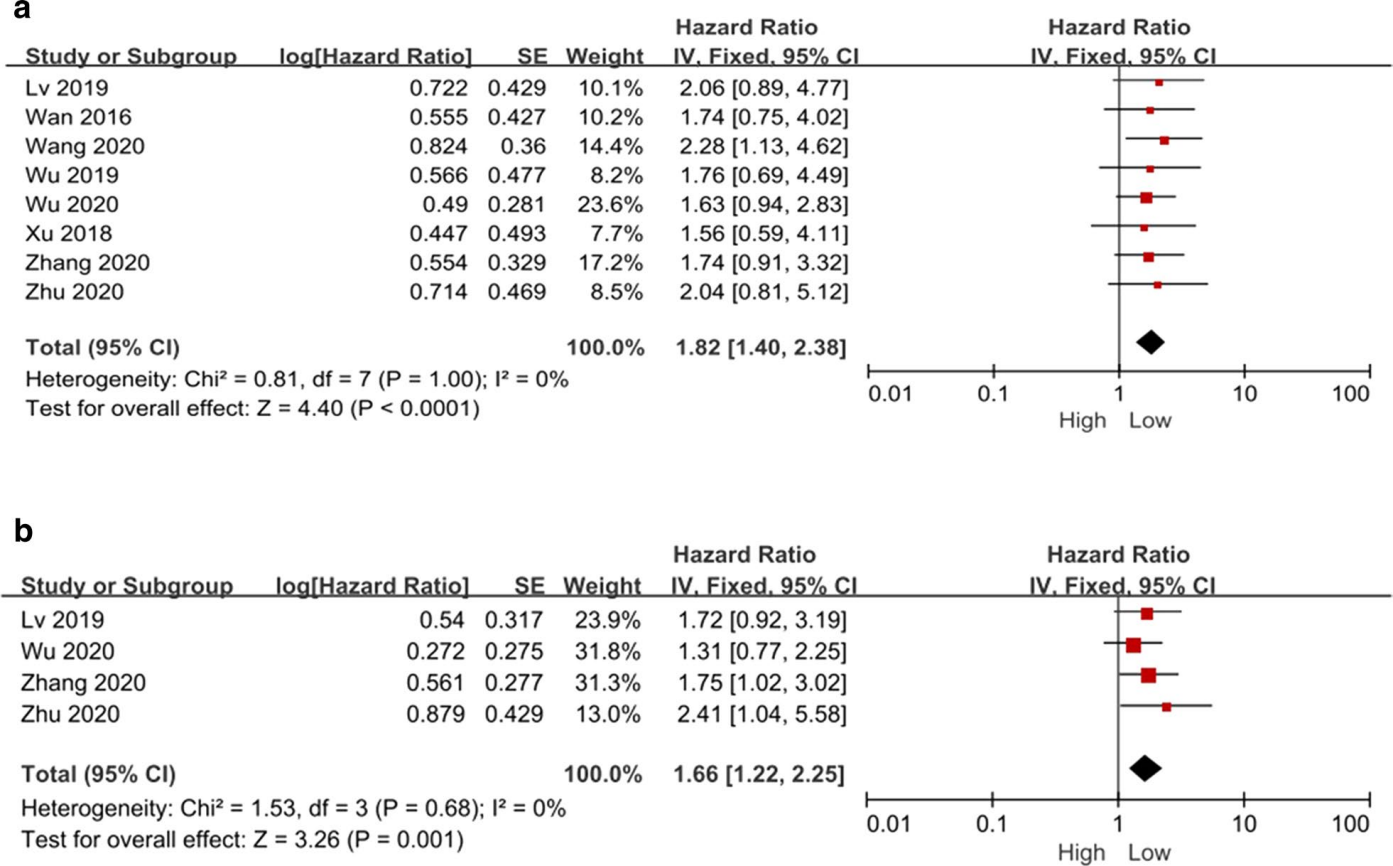
Forest plot demonstrating the relationships between lncRNA PCAT6 expression and overall survival (OS) **(A)** and progression-free survival (PFS) **(B)**

For the eight studies [17, 21, 23, 26, 27, 33–35] that reported OS, a total of 656 patients were included. Since no statistical heterogeneity was observed between these studies (*P* = 1.00, *I*^*2*^ = 0%), a fixed-effects model was used for statistical analysis. The results revealed a significant correlation between high PCAT6 expression and a lower OS (HR = 1.82, 95% CI: [1.40, 2.38], *P* < 0.0001, Fig. 2A).

For the eight studies [23, 26, 34, 35] that reported the DFS of patients, no statistical heterogeneity was observed between studies (*P* = 0.68, *I*^*2*^ = 0%), and a fixed-effect model was used for statistical analysis. The results revealed a significant correlation between high PCAT6 expression and a lower PFS (HR = 1.66; 95% CI, [1.22, 2.25]; *P* < 0.0001; Fig. 2B).

#### Relationship between PCAT6 expression and clinicopathological parameters

**Fig. 3:**
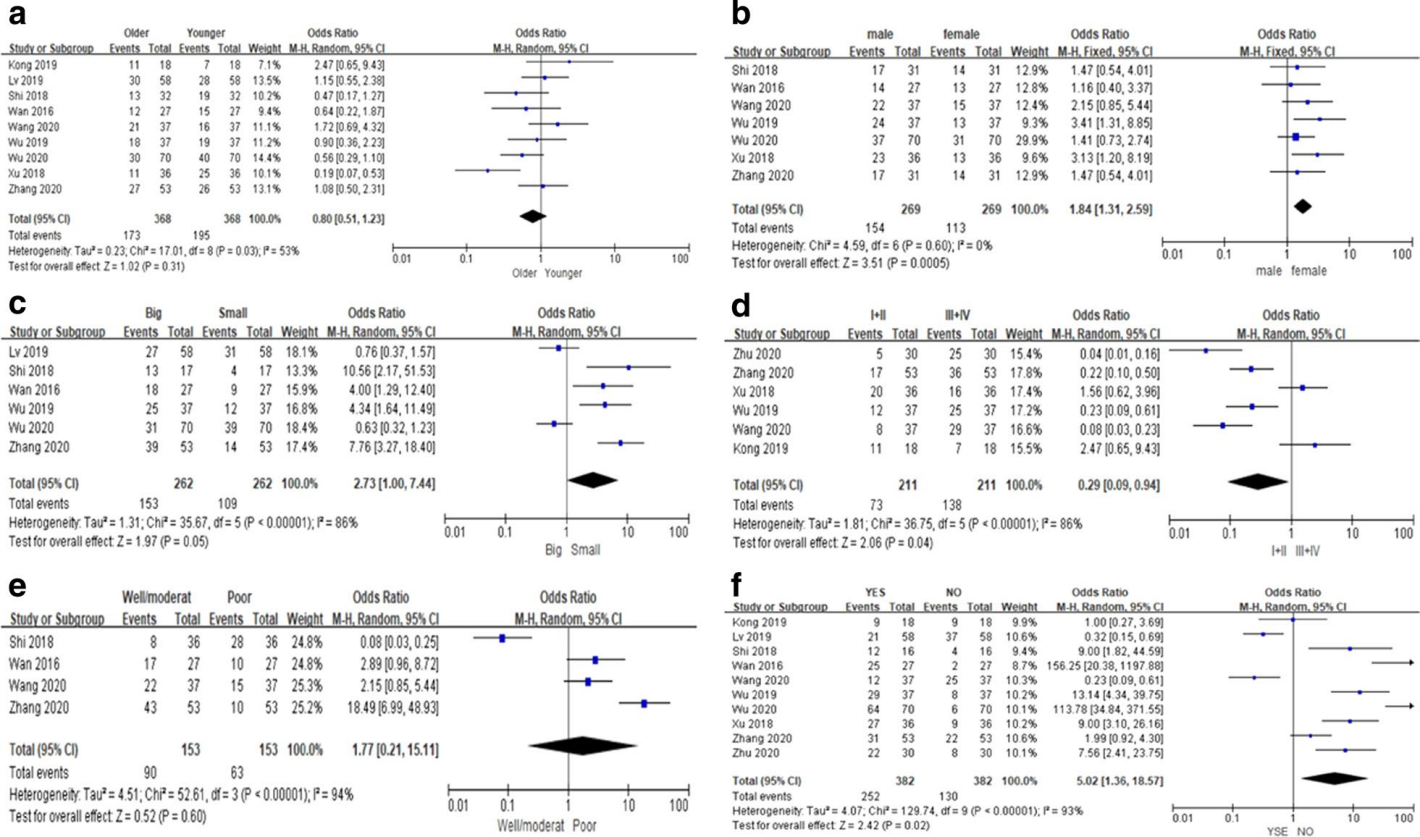
Forest plot demonstrating the relationships between lncRNA PCAT6 overexpression and **A** age, **B** gender, **C** tumor size, **D** TNM stage, **E** tumor differentiation, and **F** metastasis

All 10 included studies reported clinicopathological parameters of PCAT6 overexpression. These results are shown in Table 2. Nine studies [17, 21, 23–27, 33, 34] reported the relationship between PCAT6 overexpression and age, but no significant correlation was observed (OR = 0.80; 95% CI, [0.51, 1.23]; *P* = 0.31; Fig. 3A). Seven studies [17, 21, 23, 25, 27, 33, 34] reported the relationship between PCAT6 overexpression and gender, and a significant correlation was observed between male sex and PCAT6 overexpression (OR = 1.84; 95% CI, [1.31, 2.59]; *P* = 0.0005, Fig. 3B). Six studies [17, 21, 23, 25, 26, 34] reported the correlation between PCAT6 overexpression and tumor size. Although a correlation was observed between PCAT6 overexpression and a larger tumor size, the results were not statistically significant (OR = 2.73; 95% CI, [1.00, 7.44]; *P* = 0.05; Fig. 3C). Six studies [21, 24, 27, 33–35] reported the relationship between PCAT6 overexpression and TNM stage. A significant correlation was observed between PCAT6 overexpression and TNM stage, and there was a significant correlation between PCAT6 overexpression and clinically advanced stage (III/IV) (OR = 0.29; 95% CI, [0.09, 0.94]; *P* = 0.04; Fig. 3D). Four studies [17, 25, 27, 34] reported a relationship between the degree of tumor differentiation and high PCAT6 expression, however, no significant correlation was observed (OR = 1.77; 95% CI, [0.21, 15.11]; *P* = 0.60; Fig. 3E). All 10 studies reported a correlation between high PCAT6 expression and the presence of metastasis, and the combined results showed a significant correlation between high PCAT6 expression and metastasis (OR = 5.02; 95% CI, [1.36, 18.57]; *P* = 0.02; Fig. 3F).

**Table 2:** Meta-analysis of the studies reporting the association between over-expressed PCAT6 and clinicopathological parameters

Clinicopathological parameters	Studies	Patients	Mode	OR (95% CI)	P value	Heterogeneity	
						I^2^ (%)	P-value
Age (old vs young)	9	736	Random	0.80 [0.51, 1.23]	0.31	53	0.03
Gender (male vs female)	7	267	Fixed	1.84 [1.31, 2.59]	0.0005	0	0.60
Tumor size (big vs small)	6	524	Random	2.73 [1.00, 7.44]	0.05	86	< 0.00001
TNM stage (I + II VS III + IV)	6	211	Random	0.29 [0.09, 0.94]	0.04	86	< 0.00001
Differentiation (well + moderat vs poor)	4	306	Random	1.77 [0.21, 15.11]	0.60	94	< 0.00001
Metastasis (yse vs no)	10	746	Random	5.02 [1.36, 18.57]	0.02	93	< 0.00001

*Random* Random-effects, *Fixed* Fixed-effects, *OR* Odds ratio, *CI* confidence interval

#### Subgroup analysis

**Fig. 4:**
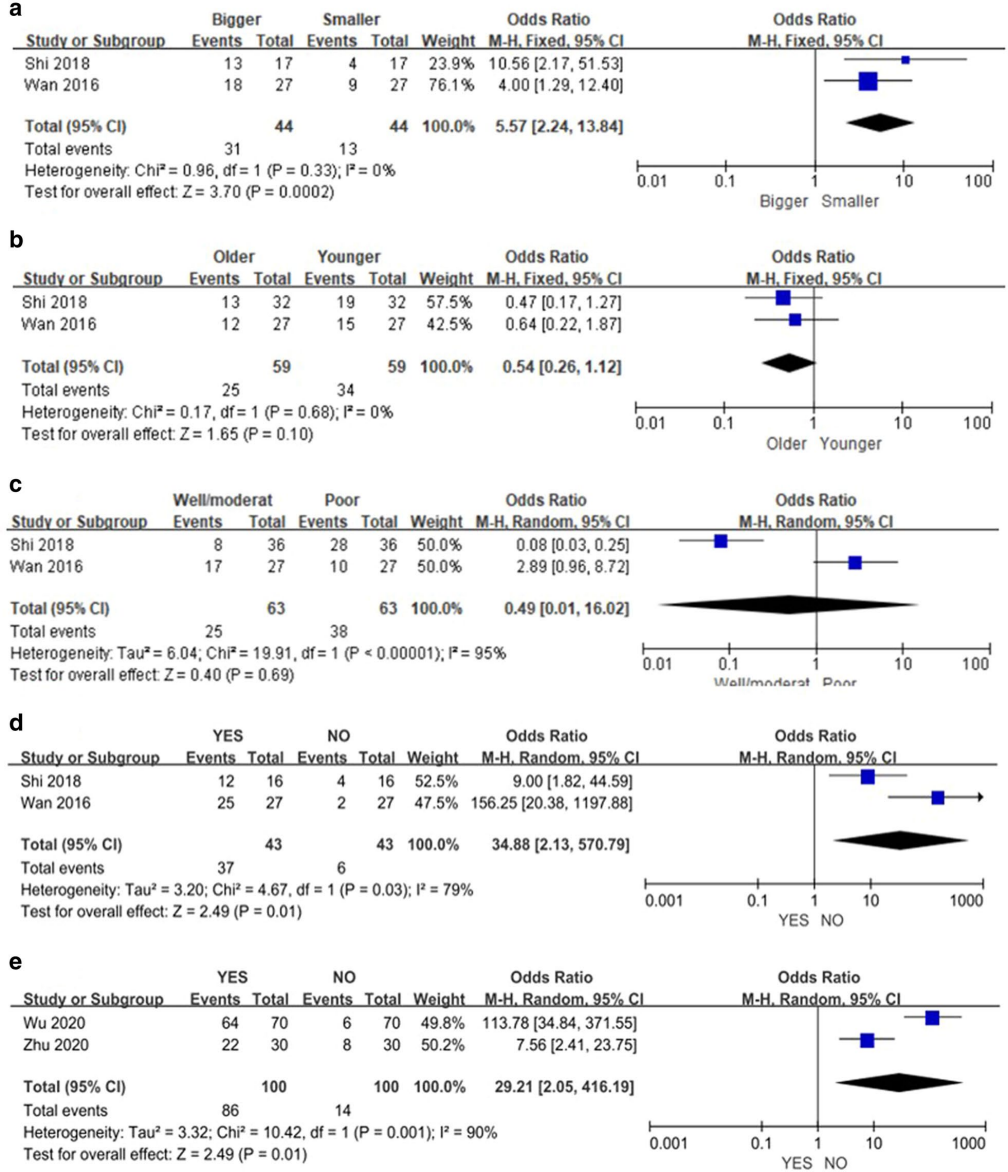
Forest plot demonstrating the relationships between lncRNA PCAT6 overexpression and **A** tumor size of lung cancer patients, **B** age of lung cancer patients, **C** degree of tumor differentiation of lung cancer patients, **D** tumor metastasis status of lung cancer patients, and **E **tumor metastasis status of osteosarcoma patients

Because of the high heterogeneity in the results concerning clinicopathological parameters, subgroup analysis was performed to identify sources of heterogeneity basesd on the cancer type. Two studies [17, 25] reported correlations between high PCAT6 expression and tumor size, age, degree of differentiation, and metastasis in lung cancer. A significant correlation between high PCAT6 expression and larger lung tumors was observed (OR = 5.57; 95% CI, [2.24, 13.84]; *P* = 0.0002; Fig. 4A), with no heterogeneity between studies (*P* = 0.33, *I*^*2*^ = 0%). No correlation was observed between high PCAT6 expression and the age of lung cancer patients (OR = 0.54; 95% CI, [0.26, 1.12]; *P* = 0.10; Fig. 4B), with no heterogeneity between studies (*P* = 0.68; *I*^*2*^ = 0%). Although a correlation between high PCAT6 expression and lower differentiation of lung cancer was observed (OR = 0.49; 95% CI, [0.01, 16.02]; *P* = 0.69; Fig. 4C), there was high heterogeneity between studies (P < 0.00001, I^2^ = 95%). Likewise, although a significant correlation between high PCAT6 expression and metastasis of lung cancer was observed (OR = 34.88; 95% CI, [2.13, 570.79]; *P* = 0.01; Fig. 4D), the data showed high heterogeneity (*P* = 0.03, *I*^*2*^ = 79%). In addition, two studies [23, 35] reported a correlation between high PCAT6 expression and metastatic osteosarcoma. Although a significant correlation between high PCAT6 expression and metastatic osteosarcoma was observed (OR = 29.21; 95% CI, [2.05, 416.19]; *P* = 0.01; Fig. 4E), there was significant heterogeneity between studies (*P* = 0.001, *I*^*2*^ = 90%).

#### Sensitivity analysis

**Fig. 5:**
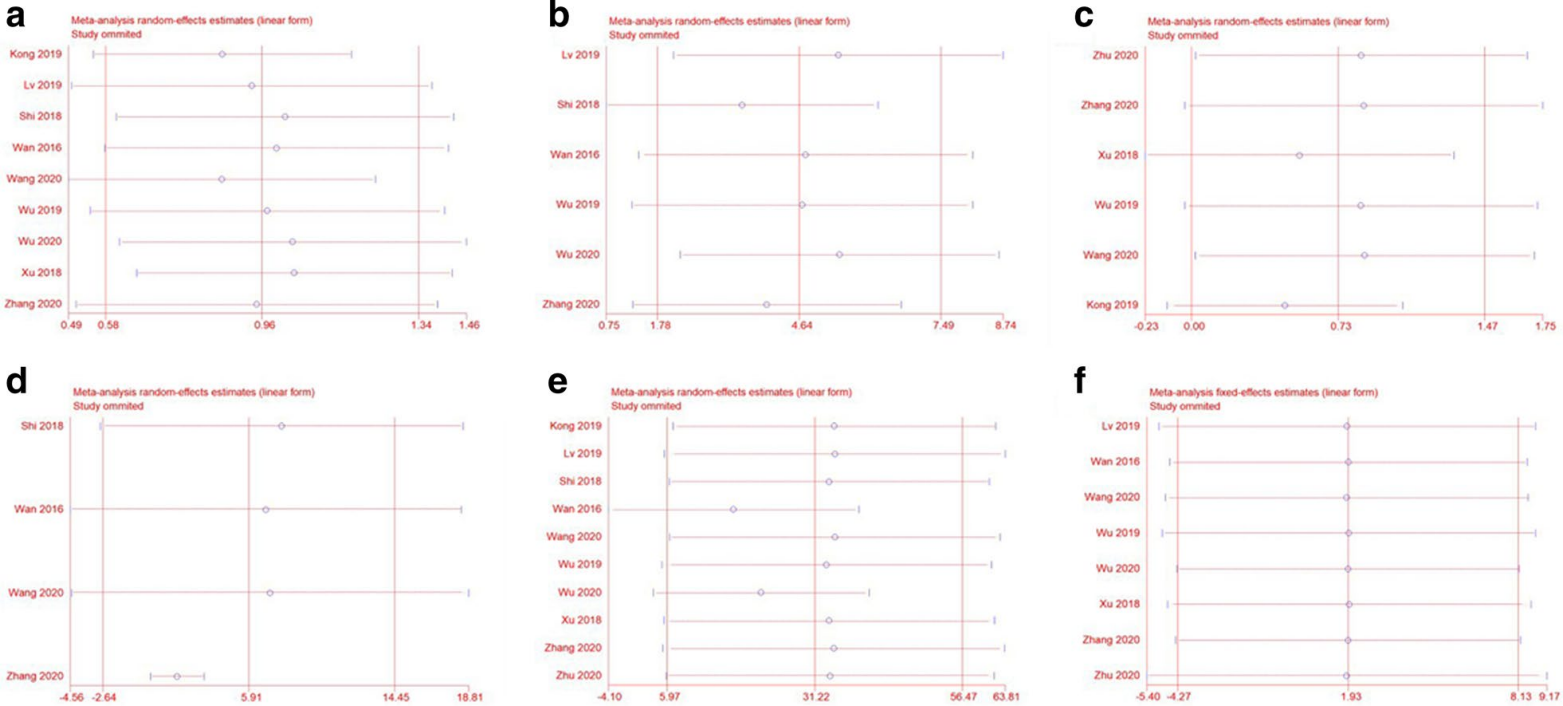
Sensitivity analysis for the meta-analysis of the association between lncRNA PCAT6 overexpression and **A** age, **B** tumor size, **C** TNM stage, **D** tumor differentiation, and **E** metastasis. Sensitivity analysis for the meta-analysis of the association between lncRNA PCAT6 expression and overall survival (OS) **(F)**

To assess the stability of the combined results, we performed a sensitivity analysis of the correlation between PCAT6 expression and OS. The sensitivity analysis showed that no individual study changed the combined results, and therefore, the OS results were considered reliable (Fig. 5F). Sensitivity analysis was also performed on the clinicopathological parameters results (Fig. 5A–E). Sensitivity analysis of the correlation between high PCAT6 expression and age showed a significant reduction in heterogeneity after excluding the studies of Kong et al. [24] and Xu et al. [33] (*P* = 0.38, *I*^*2*^ = 6%), however, no significant effect was observed on the final results (OR = 0.86; 95% CI, [0.63, 1.17]; *P* = 0.34). In the sensitivity analysis on tumor size, it was found that excluding the studies of Lv et al. [26] and Wu et al. [23] completely eliminated heterogeneity (*P* = 0.63, *I*^*2*^ = 0%), and there was a significant correlation between high PCAT6 expression and tumor size (OR = 5.86; 95% CI, [3.46, 9.90]; *P* < 0.00001). Moreover, sensitivity analysis of the correlation between high PCAT6 expression and TNM staging suggested that the study by Xu et al. [33] may be the source of heterogeneity. However, exclusion of this study did not completely eliminate heterogeneity, suggesting more robust results. Among the outcome indicators regarding the degree of tumor differentiation, studies performed by Shi et al. [25] and Zhang et al. [34] were found to be sources of heterogeneity, and after excluding these two studies, heterogeneity was eliminated (*P* = 0.69, *I*^*2*^ = 0%), thereby revealing a significant correlation between better differentiation and high expression of PCAT6 (OR = 2.43; 95% CI, [1.20, 4.94]; *P* = 0.01). During the sensitivity analysis of the correlation between high PCAT6 expression and tumor metastasisis, the study by Wan et al. [17] was identified as a source of heterogeneity. However, exclusion of this study did not eliminate heterogeneity and had no significant effect on the final results, suggesting more robust results.

#### Analysis of publication bias

**Fig. 6:**
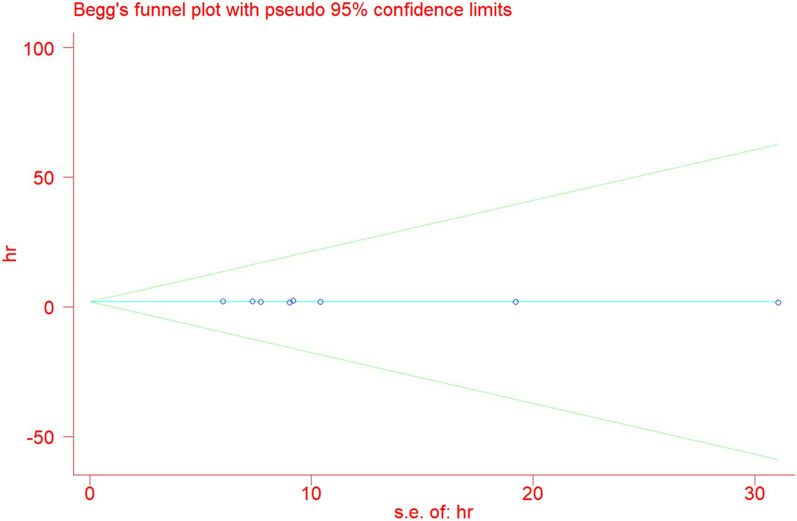
Begg’s publication bias plots of overall survival (OS)

The publication bias for PCAT6 expression and OS was analyzed using a Begg's funnel plot (Fig. 6). No obvious publication bias was observed in the included studies. Begg's test results (Pr >|z|= 0.902) revealed no publication bias.

#### Validation of PCAT6 expression in public databases

**Fig. 7:**
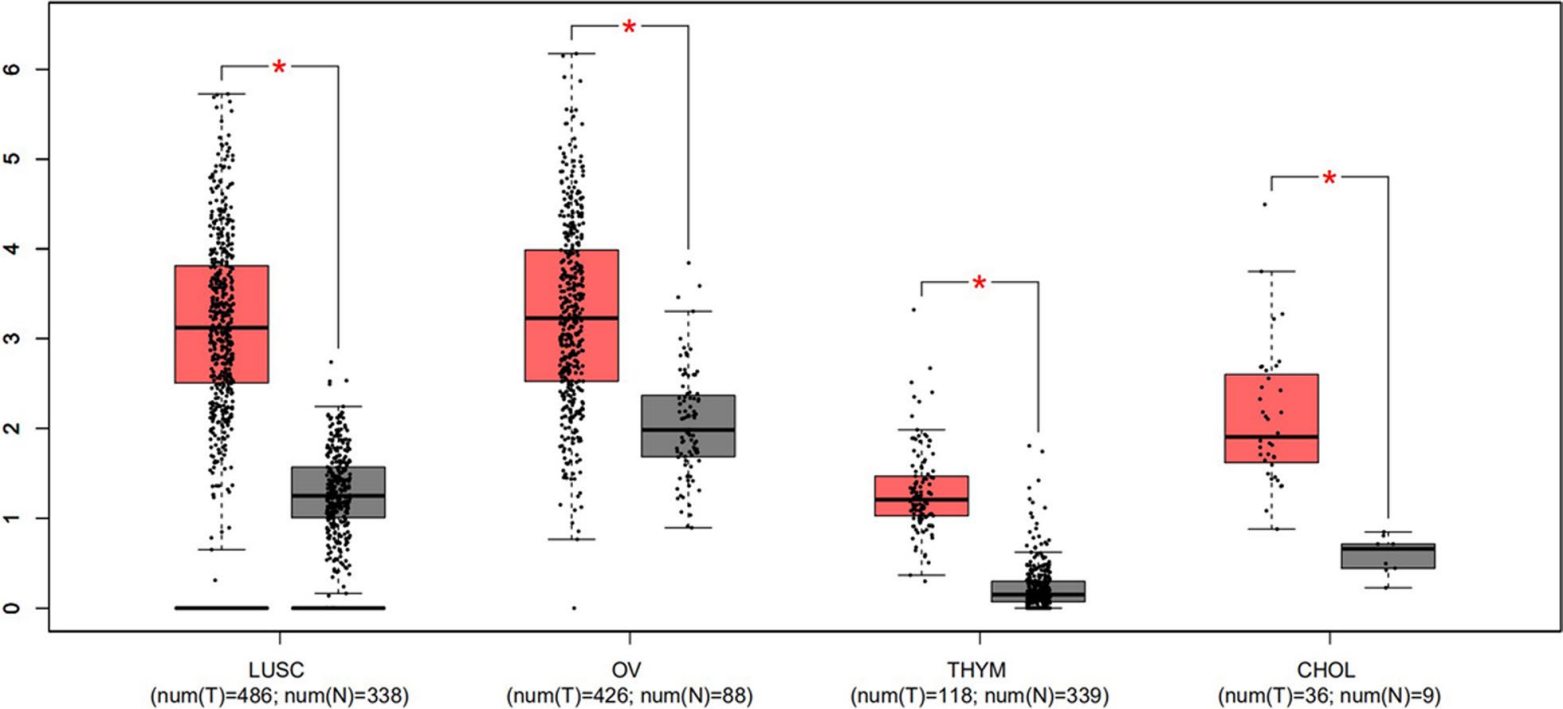
PCAT6 expression levels in tissues of four types of cancer and normal tissues. “*”, |Log_2_FC|> 1 and P < 0.01. Red box plots represent PCAT6 expression in cancer tissues, grey box plots represent PCAT6 expression in normal tissues

**Fig. 8:**
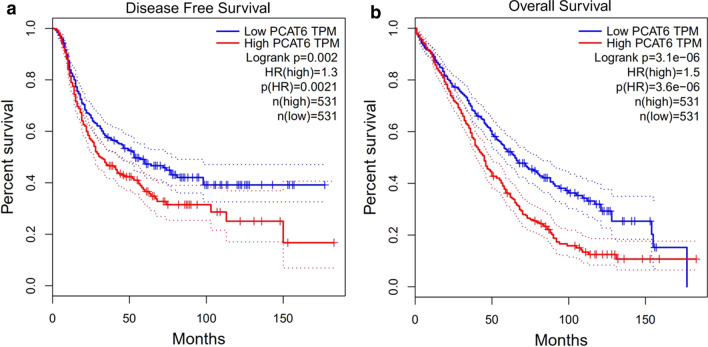
The relationship between PCAT6 expression and cancer patient prognosis in the GEPIA cohort. **A** Overall survival (OS) plots based on PCAT6 expression in four types of cancer (n (low) = 531 vs n (high) = 531). **B** Disease-free survival (DFS) plots based on PCAT6 expression in four types of cancer (n (low) = 531 vs n (high) = 531)

To further validate our results, PCAT6 expression was evaluated in nine cancers using GEPIA clinical data. The results revealed that PCAT6 was up-regulated in most cancers, including Cholangio carcinoma (CHOL), lung squamous cell carcinoma (LUSC), ovarian serous cystadenocarcinoma (OV), and thymoma (THYM) (Fig. 7). Furthermore, high expression of PCAT6 indicated poor patient prognosis. The prognostic data for four cancers (CHOL, LUSC, OV, and THYM) were combined. Next, 1062 patients with nine cancer types were divided into two groups based on median levels of PCAT6 expression. OS and DFS were shorter in the PCAT6 high expression group compared to the PCAT6 low expression group, thereby confirming that PCAT6 overexpression was associated with poor OS in various human cancers (*P* < 0.0001) (Fig. 8). These results are similar to the results obtained in our present study.

We also used NGDC to search and validate the expression of PCAT6 in various cancers. Finally, we validated PCAT6 expression in various cancers in lnCAR, an online analysis site based on Gene Expression Omnibus (GEO) [36]. A total of 30 datasets were collected, representing data from a total of six types of cancer (bladder cancer, breast cancer, colorectal cancer, lung cancer, prostate cancer, and ovarian cancer). In comparison with normal tissue, PCAT6 expression was increased in all included cancer tissues, and the observed differences were statistically significant (*P* < 0.05). The results are shown in Table 3.

**Table 3:** Differential expression of PCAT6 in different cancers

Tumor Type	GSE number	Sample Number		Expression	P-value
		Tumor	Normal		
Bladder cancer	GSE13570	188	68	High	0.0000
	GSE89006	4	4	High	0.0271
	GSE7476	9	3	High	0.0068
	GSE37817	18	6	High	0.0057
	GSE52519	9	3	High	0.0323
	GSE76211	3	3	High	0.0892
Breast cancer	GSE42568	104	17	High	0.0004
	GSE45827	130	11	High	0.0007
	GSE50428	26	5	High	0.0000
	GSE65194	163	11	High	0.0016
Breast cancer (Invasive ductal carcinoma)	GSE10780	42	143	High	0.0000
	GSE10810	22	22	High	0.0004
	GSE21422	5	5	High	0.0003
	GSE40206	73	73	High	0.0000
Breast cancer (Inflammatory breast cancer)	GSE17907	21	4	High	0.0185
Colorectal cancer	GSE21510	123	25	High	0.0000
	GSE21815	132	9	High	0.0000
	GSE37364	27	38	High	0.0000
	GSE39582	566	19	High	0.0001
	GSE83889	101	35	High	0.0008
Lung cancer	GSE27262	25	25	High	0.0000
	GSE89039	8	8	High	0.0000
Lung cancer (Non-small cell)	GSE101929	32	34	High	0.0000
Prostate cancer	GSE8511	25	16	High	0.0004
	GSE32571	59	39	High	0.0002
	GSE38241	18	21	High	0.0000
	GSE68555	66	83	High	0.0000
Ovarian cancer	GSE18520	53	10	High	0.0003
	GSE40595	31	8	High	0.0001
	GSE54388	16	6	High	0.0047

#### Molecular mechanisms of PCAT6 oncogenesis in various cancers

The known molecular mechanisms of PCAT6 oncogenesis in various cancers are summarized below.

- In lung cancer, PCAT6 combined with EZH2 to reduce LATS2 promoter activity (via H3K27 trimethylation in the LATS2 promoter region) and regulated miR-330-5p, thereby affecting proliferation and metastasis of non-small cell lung cancer (NSCLC) cells. In addition, PCAT6 affected the biological function of lung cancer cells by modulating the expression of two cancer-related proteins, c-Myc and p53 [17, 25, 37].
- In osteosarcoma, PCAT6 promoted the progression of osteosarcoma by sponging miR-185-5p to activate the TGF-β pathway, by regulating the miR-143-3p/ ZEB1 pathway, and promoting the expression of MDM2, and by inhibiting the expression of p53 and p21 [35, 38].
- In bladder cancer, PCAT6 promoted the progression of bladder cancer cells by targeting miR-513a-5p [39].
- In breast cancer, PCAT6 promoted Triple-Negative Breast Cancer (TNBC) cell proliferation, migration, invasion, epithelial-mesenchymal transition (EMT), and angiogenesis, as well as tumor growth and metastasis, via upregulation of VEGFR2 by sponging miR-4723-5p and recruitment of USP14 [40].
- In gastric cancer, PCAT6 promoted the proliferation and metastasis of gastric cancer cells by endogenously upregulating MKRN3 expression in competition with miR-30 [33].
- In ovarian cancer, PCAT6 promoted the development and progression of ovarian cancer by regulating PTEN. In vitro experiments also revealed that suppression of PTEN expression counteracted the role of PCAT6 in ovarian cell carcinoma [24].
- In cervical cancer, PCAT6 promoted the progression of cervical cancer by regulating the Wnt/ β-catenin pathway, secreting miR-543, and by upregulating ZEB1 [26, 41].
- In cholangiocarcinoma, PCAT6 induces cell proliferation and invasion by decreasing miR-330-5p expression in cholangiocarcinoma cells [42].
- In glioblastoma, PCAT6 upregulates IGF2BP1 expression via miR-513, forming a PCAT6/ miR-513/ IGF2BP1 positive feedback loop, and thus promoting glioblastoma progression [43].
- In pancreatic ductal carcinoma, PCAT6 promoted the growth of pancreatic ductal carcinoma by secreting miR-185-5p to upregulate the expression of the cancer-related gene CBX2 [27].
- In intestinal mesenchymal tumors, PCAT6 promoted proliferation of gastrointestinal mesenchymal tumor cells by upregulating PRDX5 via miR-143-3p [44].
- In pituitary adenomas, PCAT6 may promote pituitary adenoma progression in vitro and in vivo by targeting the miR-139-3p/ BRD4 axis [45].

The details of these molecular mechanisms are summarized in Table 4.

**Table 4:** Molecular mechanisms of PCAT6 oncogenesis in various cancers

Tumor type	Expression	Molecular mechanisms	References
Ovarian cancer	High	Promotes ovarian carcinogenesis and progression by regulating PTEN, while suppression of PTEN expression counteracts the role of PCAT6 in ovarian cell carcinoma	[23]
Cervical cancer	High	Promotes proliferation and metastasis of cervical cancer cells through regulation of the Wnt/ β-catenin pathway	[26]
Colon cancer	High	Promote apoptosis in colon cancer cells by regulating the level of anti-apoptotic protein ARC	[46]
Lung cancer	High	Reduces LATS2 promoter activity by binding to EZH2 leading to H3K27 trimethylation in the LATS2 promoter region, thereby affecting the proliferation and metastasis of non-small cell lung cancer cells	[25]
	High	Influences the biological function of lung cancer cells by affecting the expression of p53 and c-Myc, key proteins that regulate cancer progression	[17]
	High	Promotes proliferation, migration, and invasion of non-small cell lung cancer cells through regulation of miR-330-5p	[37]
Pancreatic ductal carcinoma	High	Promotes the proliferation, migration, and invasion of pancreatic ductal carcinoma cells by secreting miR-185-5p to upregulate the expression of the cancer-related gene CBX2	[27]
Osteosarcoma	High	Progression of osteosarcoma is accelerated by regulating the miR-143-3p/ ZEB1 pathway	[23]
	High	Activation of TGF-β pathway promotes osteosarcoma progression by sponging miR-185-5p and upregulating the expression of TGFBR1 and TGFBR2	[35]
	High	Promotes proliferation, migration, and invasion of osteosarcoma cells by suppressing p53 and p21 expression through promotion of MDM2 expression	[33]
Stomach cancer	High	Promotes proliferation and metastasis of gastric cancer cells by endogenously competing with miR-30 to upregulate MKRN3 expression	[39]
Bladder cancer	High	Promotes the progression of bladder cancer cells by targeting miR-513a-5p	[43]
Glioblastoma	High	Upregulates IGF2BP1 expression by miR-513, by forming a PCAT6/ miR-513/ IGF2BP1 positive feedback loop, thus promoting the progression of glioblastoma	[44]
Gastrointestinal mesenchymal tumor	High	Promotes proliferation of gastrointestinal mesenchymal tumor cells through miR-143-3p upregulation of PRDX5	[47]
Breast cancer	High	Promotes TNBC cell proliferation, migration, invasion, EMT, and angiogenesis, as well as tumor growth and metastasis via upregulation of VEGFR2 through sponging miR-4723-5p and recruiting USP14	[40]
	High	Silencing PCAT6 inhibits the proliferation of triple-negative breast cancer cells and promoted apoptosis by upregulating the expression of miR-185-5p and downregulating the expression of TPD52	[38]
Bile duct cancer	High	Inhibits cell proliferation and invasion by reducing miR-330-5p expression in bile duct cancer cells	[42]
Pituitary adenoma	High	Promotes pituitary adenoma progression by targeting the miR-139-3p/ BRD4 axis	[45]

## Discussion

With the rapid increase of cancer incidence and mortality worldwide, cancer is set to become the leading cause of death, and a significant impediment to increasing life expectancy in all countries this century [46]. In many cancers, over-activation or overexpression of anti-apoptotic proteins is frequently observed, and an imbalance between cell growth and apoptosis is an integral part of the oncogenic process of tumors, which makes tumor treatment challenging [47]. Although in recent decades cancer treatment has notably progressed, the prognosis for most cancer types remains poor. Therefore, early diagnosis and treatment are important to improve the prognosis in cancer patients. However, the sensitivity and specificity of currently used tumor markers are not satisfactory [40].

LncRNAs are transcribed by RNA polymerase II, and their expression is tissue-specific and highly conserved within mammals. Numerous studies have demonstrated that lncRNAs are involved in a range of biological processes, including X-chromosome silencing, chromatin modification, transcriptional interference and activation, and intranuclear translocation by regulating gene expression in the form of RNA, lncRNAs are involved in almost all human physiological and pathological processes [24]. Recently, the field of lncRNAs has attracted significant attention, and numerous studies have revealed that PCAT6 expression is up-regulated in various cancers, and that PCAT6 has the potential to become a new diagnostic biomarker and therapeutic target.

Kong et al. [24] found that PCAT6 expression was higher in ovarian cancer tissues than in paraneoplastic tissues, and that high PCAT6 expression was strongly associated with poor prognosis in ovarian cancer patients. Shi et al. [45] demonstrated an increased abundance of PCAT6 in TNBC tissues and cells, and found that PCAT6 levels positively correlated with lymph node metastasis and the clinical stage. Shi et al. [25] and Wan et al. [17] both reported that PCAT6 expression was upregulated in lung cancer (in comparison with normal lung tissue), and that PCAT6 expression correlated with the tumor size, lymph node metastasis, and TNM stage. Wang et al. [27] reported that PCAT6 expression was upregulated in pancreatic ductal carcinoma, and that PCAT6 expression correlated with TNM stage, the presence of lymph node metastasis, and OS of patients with pancreatic ductal carcinoma. Wu et al. [23] and Zhu et al. [35] both reported that PCAT6 expression was significantly higher in osteosarcoma tissues (in comparison with normal bone tissues), and that high expression of PCAT6 closely associated with poor prognosis in patients with osteosarcoma. Xu et al. [33] showed that PCAT6 expression was higher in gastric cancer tissues (compared with normal adjacent tissues), and that PCAT6 expression correlated with TNM stage, lymph node metastasis, and OS in gastric cancer patients. Finally, Zhang et al. [34] and Huang et al. [47] both reported that PCAT6 expression was higher in colon cancer tissues (in comparison with adjacent normal tissues), and that the high expression of PCAT6 closely associated with poor patient prognosis.

The aim of the present study was to elucidate the relationship between PCAT6 and cancer, clinical case characteristics, and prognosis. In our meta-analysis, PCAT6 overexpression significantly correlated with a lower OS and PFS, and PCAT6 overexpression significantly reduced OS and PFS in patients. Validation using TCGA database results confirmed our findings that PCAT6 overexpression significantly correlated with a shorter OS and DFS. Regarding the correlation between PCAT6 overexpression and clinicopathological parameters, our results revealed that men were more likely to show high expression of PCAT6 (one possible explanation is that PCAT6 is associated with sex chromosomes, although this has not been reported). Regarding the correlation between PCAT6 overexpression and tumor size, PCAT6 overexpression and a larger tumor volume significantly correlated (after excluding cervical cancer and osteosarcoma). However, no heterogeneity was observed between the included studies, the results of subgroup analysis revealed that a larger tumor volume and PCAT6 overexpression significantly correlated in lung cancer. As no heterogeneity was observed between the included studies, therefore, it can be concluded that PCAT6 overexpression and a larger tumor volume were associated in lung, colorectal, and bladder cancers. Regarding the correlation between PCAT6 overexpression and TNM stage, a negative correlation was observed between PCAT6 expression and advanced tumor stage (III + IV).

No significant correlation was observed between PCAT6 overexpression and the degree of tumor differentiation. Moreover, subgroup analysis revealed that PCAT6 overexpression did not significantly correlate with the degree of differentiation of lung cancer. These results contrast with the data presented by Shi et al. [25] and Wan et al. [17], who reported completely different results with lung cancer. This difference may be due to the different types of lung cancer included in these two studies. Shi et al. [25] included patients with non-small cell lung cancer, while Wan et al. [17] did not specify the specific lung cancer type of the patients included.

To further investigate PCAT6 expression in various cancers, we used two public databases to determine PCAT6 expression in various cancers. The results revealed that PCAT6 expression was significantly higher in eight cancers, and that high PCAT6 expression strongly associated with poor patient prognosis. These results validated the findings presented in this study.

In the present study, we first explored the role of PCAT6 in tumors and found a significant correlation between PCAT6 overexpression and cancer metastasis. Because of high heterogeneity, we performed subgroup and sensitivity analyses, and these results revealed a significant correlation between PCAT6 overexpression and tumor metastasis in both lung cancer and osteosarcoma. The heterogeneity remained high, therefore, further studies are needed to validate our findings. Several limitations of our study should be considered. First, all studies were from the People's Republic of China. Furthermore, only a small number of cases were included, and sample sizes were low for some cancer types. Therefore, the possibility arises that our results apply only to Asian patients. To address this limitation, we subsequently validated our results using public databases, providing support for our conclusions. Second, the HR for OS and PFS as extracted from Kaplan–Meier curves, potentially leading to errors. Third, we offer no standard cut-off value for overexpression and under-expression of PCAT6. Finally, because the included studies were all in English, some studies may have been omitted [48].

## Conclusion

We found that PCAT6 overexpression was significantly correlated with shorter OS and PFS in cancer patients. In addition, a significant correlation was observed between PCAT6 overexpression and individual tumor clinicopathological parameters, including TNM stage, gender, and tumor metastasis. However, PCAT6 overexpression was not associated with patient age and tumor differentiation. In summary, PCAT6 may be useful as a novel tumor predictor, and the critical role of PCAT6 in human cancers should be explored further. To confirm these results and validate their clinical significance, large-scale and multicenter studies will be needed.

## Data Availability

All data are included in this manuscript.

## Abbreviations

lncRNA: Long noncoding RNA
PCAT6: Prostate cancer-associated transcript 6
HR: Hazard ratio
OR: Odds ratio
OS: Overall survival
DFS: Disease-free survival
PFS: Progression-free survival
95% CI: 95% Confidence interval
qRT-PCR: Quantitative real-time polymerase chain reaction
NOS: The Newcastle–Ottawa Quality Assessment Scale
GEPIA: Gene Expression Profiling Interactive Analysis
TCGA: The Cancer Genome Atlas
CHOL: Cholangio carcinoma
LUSC: Lung squamous cell carcinoma
OV: Ovarian serous cystadenocarcinoma
THYM: Thymoma
NGDC: Database Resources of the National Genomics Data Center
